# Three-wave X-ray diffraction in distorted epitaxial structures

**DOI:** 10.1107/S0021889813011709

**Published:** 2013-06-07

**Authors:** Reginald Kyutt, Mikhail Scheglov

**Affiliations:** a Ioffe Physical Technical Institute, Russian Academy of Sciences, St Petersburg, Russian Federation

**Keywords:** X-ray diffraction, multiple diffraction, epitaxial layers, structural defects, wurtzite structure

## Abstract

Three-wave diffraction has been measured for a set of GaN, AlN, AlGaN and ZnO epitaxial layers grown on *c*-sapphire. A Renninger scan for the primary forbidden 0001 reflection was used. For each of the three-wave combinations, θ-scan curves were measured. The intensity and angular width of both ϕ- and θ-scan three-wave peaks were analyzed. The experimental data were used to determine properties of the multiple diffraction pattern in highly distorted layers. It is shown that the FWHM of θ scans is highly sensitive to the structural perfection and strongly depends on the type of three-wave combination. The narrowest peaks are observed for multiple combinations with the largest *l* index of the secondary *hkl* reflection. An influence of the type of the dislocation structure on the θ-scan broadening was revealed. These experimental facts are interpreted by considering the scanning geometry in the reciprocal space and taking into account the disc-shaped reciprocal-lattice points. The total integrated intensities of all the three-wave combinations were determined and their ratios were found to be in only a qualitative agreement with the theory. For AlGaN layers, the presence of the nonzero 0001 reflection was revealed, in contrast to AlN and GaN films.

## Abstract 2

Three-wave diffraction has been experimentally studied for a set of III–nitride and ZnO epitaxial films differing in thickness and structural perfection. Properties of the multiple diffraction pattern in highly distorted layers are analyzed.

## 1. Introduction

Multiple-wave diffraction was discovered by Renninger (1937 ▶) who performed pioneering experiments and developed two versions of the kinematic theory for analysis of the corresponding diffraction patterns. A large number of theoretical and experimental studies have been reported in this field (Cole *et al.*, 1962 ▶; Kottwitz, 1968 ▶; Prager, 1971 ▶; Chang, 1980 ▶). In a series of reports by Rossmanith (2000 ▶, 2002 ▶, 2007 ▶), a detailed analysis of multiple diffraction in a kinematic approach, with experimental verification of the theory, has been given. Her experiments were mostly performed on single crystals that, as a rule, had a spherical shape.

For epitaxial layers, the multiple-wave diffraction technique has only been employed in a few studies, with the lattice parameter and strain of the epitaxial layer found from the angular positions of multiple-wave peaks in the Renninger diagram (Chang, 1980 ▶; Sasaki *et al.*, 1996 ▶; Korytár *et al.*, 1998 ▶; Freitas *et al.*, 2007 ▶). The multiple diffraction method has not been used to study the defect structure.

Epitaxial layers of III–nitrides (GaN, AlN and InN) and a structurally close compound ZnO have a wurtzite structure and are mostly grown on sapphire substrates. In this case, the mismatch in the (0001) interface plane is rather large (13% for GaN), which leads to the generation of a high density of dislocations. The dislocation structure of layers of this kind has been studied in sufficient detail. In most cases, it is predominantly constituted by edge and screw dislocations growing along the normal to the surface (Lei *et al.*, 1993 ▶; Heying *et al.*, 1996 ▶; Metzger *et al.*, 1998 ▶; Kyutt *et al.*, 1999 ▶; Heinke *et al.*, 2001 ▶).

In X-ray diffraction studies, the dislocation structure is commonly characterized using a mosaic model with such parameters as the block size in two directions, strain in blocks, and rotation of blocks about an axis parallel to the surface (tilting) and about the normal to the surface (twisting). A more detailed characterization of nitride layers by introduction of the microdistortion tensor has been suggested by Ratnikov *et al.* (2001 ▶). Its components can be used to estimate the density of individual families of dislocations. To obtain all components of the microdistortion tensor, the θ–2θ and θ scanning modes should be used in three diffraction geometries. Ratnikov *et al.* (2001 ▶) employed for this purpose the symmetric Bragg geometry, symmetric Laue geometry, and skew or grazing diffraction. The sizes of the coherent scattering domains in two directions and the density of various dislocation ensembles are determined by analysis of the FWHM of the experimental diffraction peaks. In a simplified mosaic model, a combination of symmetric and asymmetric reflections and a skew geometry is also required (Srikant *et al.*, 1997 ▶). A transition from one geometry to another, with the corresponding tuning of a sample, is a complex experimental procedure. Each three-wave Renninger peak combines reflections in different geometries. Therefore, these peaks carry information about the broadening in different directions. This makes it possible to determine the structural state of a crystal by a simpler method, without changing the geometry of measurements. The Renninger diagrams for GaN and ZnO were first measured by Bläsing & Krost (2004 ▶), Bläsing *et al.* (2009 ▶) and von der Ahe *et al.* (2009 ▶). Bläsing & Krost (2004 ▶) calculated the positions and relative intensities of three-wave reflections on the ϕ scale and demonstrated their agreement with experimental results. Bläsing *et al.* (2009 ▶) demonstrated that the angular widths of three-wave diffraction peaks of GaN vary between layers grown in different conditions, although no detailed analysis revealing any regularities was made.

## 2. Features of the three-wave diffraction pattern, found in a previous study

Our previous studies of epitaxial films of III–nitrides have ascertained the main features of the three-wave diffraction pattern in distorted layers with the wurtzite structure (Kyutt, 2010 ▶, 2011 ▶, 2012 ▶). In each 30° angular interval of the azimuthal rotation (ϕ scan), we observed all of the ten three-wave peaks for GaN, determined by the diffraction geometry with the 0001 primary forbidden reflection and Cu *K*α radiation. It was shown that the ϕ-scan Renninger peaks are less sensitive to the degree of structural perfection, compared with the θ-mode peaks. The strongest dependence on the dislocation density for the latter peaks is observed for 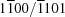 and 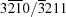 three-wave combinations with a pure Laue component of secondary reflection, whereas the 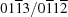 combination with a large Bragg component exhibits the weakest dependence.

For thick (>10 µm) epitaxial films, the three-beam Renninger diffraction peaks exhibit a splitting due to the large-block structure of the epilayers. In the case of ϕ scanning, the FWHM values of individual peaks vary within the range 0.15–0.40° and are almost independent of the three-wave combination. The optimal methods for precisely determining the lattice parameters *a* and *c* were shown, with parameter *a* best determined by measuring the angle between the neighboring (specularly reflected from the ϕ = 30° line) three-wave peaks of the combination 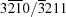.

Kyutt (2012 ▶) measured the three-beam X-ray diffraction in AlGaN/GaN superlattices. It was shown that a diffraction pattern with satellites on the θ–2θ curve of the 0001 reflection can be obtained in the azimuthal position of the three-beam diffraction.

## 3. Experimental

Three-wave diffraction is observed when, along with the reciprocal-lattice points (RLPs) 0 and *H*1 (standard two-wave diffraction), one more point *H*2 lies on the Ewald sphere. In this case, Bragg’s conditions are satisfied not only for reflections *H*1 and *H*2 with reciprocal-lattice vectors **OH**1 and **OH**2, but also, automatically, for the reflection with vector **H**1**H**2 = **OH**1 − **OH**2. The most widely used and simplest scheme for the observation of multiple diffraction is the Renninger scanning scheme. In this case, the sample is adjusted for obtaining symmetrical Bragg reflection *H*1 (incident beam with wavevector **k**_0_ and diffracted beam with **k**_1_); then, the sample remaining under these conditions is rotated about the reciprocal-lattice vector **OH**1 (or, which is the same, about the normal to the surface). For the primary reflection, we take the forbidden or very weak (quasi-forbidden) reflection. In this case, with RLP *H*2 lying on the Ewald sphere, a three-wave peak appears on the scanning diagram (dependence of the intensity in the **k**_1_ direction on the azimuthal angle ϕ). This peak was named by Renninger as *Umweganregung* because the beam for the third reflection **H**2**H**1 is incident in the **PH**2 direction and is reflected in the direction **PH**1 (= **k**1), where **P** is the center of the Ewald sphere.

The objects of study were GaN, AlN, AlGaN and ZnO epitaxial layers grown on the *c* plane of sapphire, with different thicknesses and degrees of perfection. These samples have been thoroughly examined previously by conventional two-wave diffractometry.

For the primary reflection, we used the 0001 reflection forbidden for wurtzite structures. The Renninger diagrams (ϕ scan) were measured for each sample in rough (with a ϕ step of 0.5° in an angular interval of 120°) and precision versions (with a step of 0.03° in an angular interval of 30°). For each of the three-wave peaks observed in the Renninger diagram, the θ-scan curves were also measured (rotation of the sample in an invariable azimuthal position). Measurements were made with Cu *K*α radiation in a double-crystal configuration of the diffractometer, with a Ge crystal in 111 reflection as the monochromator. The primary intensity (after the monochromator) was *I*_0_ = 4 × 10^6^ counts per second.

Since the shape of the peaks on the Renninger diagram was rather intricate and the θ-scan curves had a single symmetric peak, the integrated intensity of three-wave reflections was calculated as the area under the ϕ-scan curve, multiplied by the FWHM of the corresponding peak of the θ mode. Thus, the dimension of the integrated intensity in our case is counts per second per square degree.

## 4. Results and discussion

### 4.1. Renninger scan for ZnO and AlN

Table 1 ▶ lists the azimuthal angular positions of all the possible three-wave peaks, calculated for GaN, AlN and ZnO. The origin ϕ = 0 corresponds to the azimuthal direction [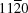] lying in the plane of scattering. In our calculations, we used the following values of the lattice parameters *c* and *a*: 5.1851 and 3.189 Å for GaN, 4.9816 and 3.113 Å for AlN, and 5.2066 and 3.2498 Å for ZnO. According to geometric considerations, the pattern comprises ten three-beam diffraction peaks (for GaN and AlN) within a 30° angular interval, which are mirror-reflected at ϕ = 30°, and then this combination is repeated periodically every 60°. ZnO has the larger parameter *a*, so the Renninger diagram contains two more peaks for the following reflections: 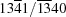 and 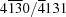.

**Figure 1:**
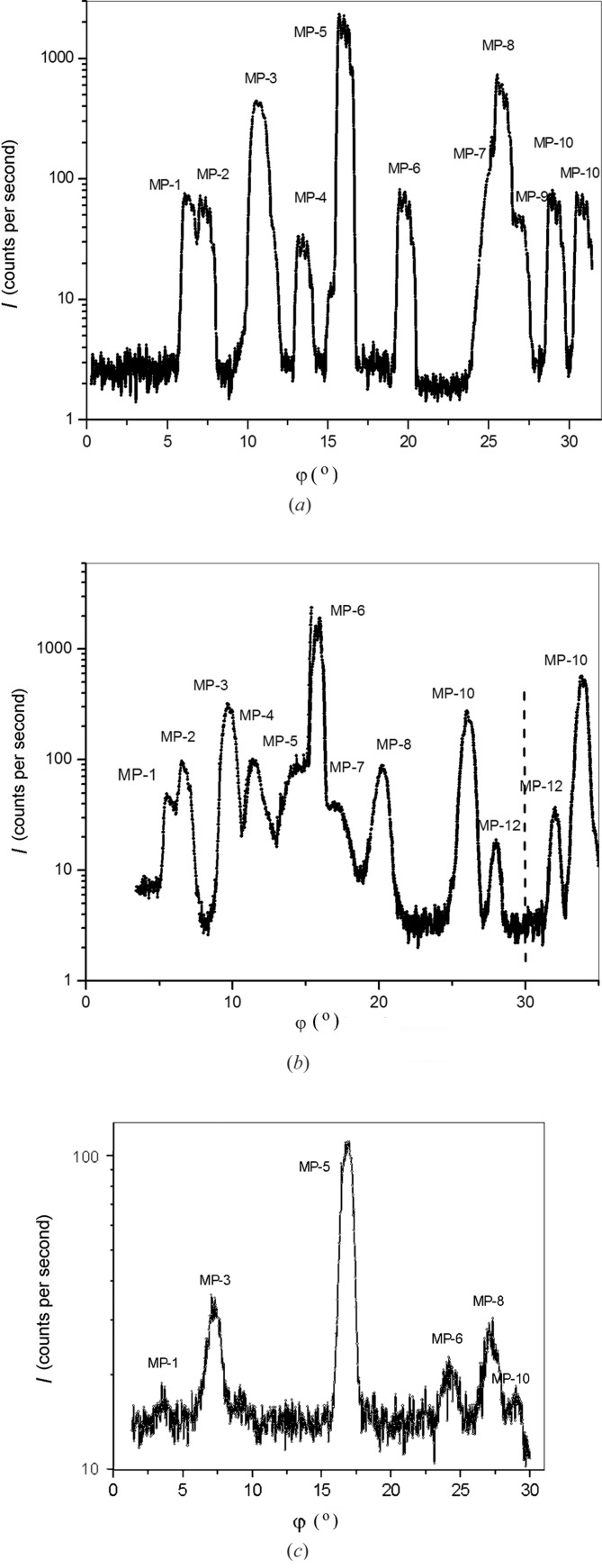
Renninger scans for (*a*) GaN (*t* = 20 µm), (*b*) ZnO (*t* = 4 mm) and (*c*) AlN (*t* = 1.2 µm) epitaxial layers. The peak designations are the same as those in Table 1 ▶.

Fig. 1 ▶ shows examples of the typical Renninger scans for GaN, AlN and ZnO epilayers. The observed peaks are identified as indicated in Table 1 ▶. In the angular interval 0–30°, ten three-wave peaks for GaN and 12 peaks for ZnO can be clearly seen. Their angular positions agree with the results of calculations, except for the peak designated in Fig. 1 ▶(*a*) as MP-4. Its position does not correspond to the 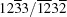 combination, which must give a peak at an angle smaller by 2° [according to our calculation and to that by Bläsing & Krost (2004 ▶)]. For AlN, thin layers were under study and, therefore, we observe only the highest intensity three-wave peaks in Fig. 1 ▶(*c*).

### 4.2. Broadening of θ-scan peaks. Comparison with two-wave diffraction

**Figure 2:**
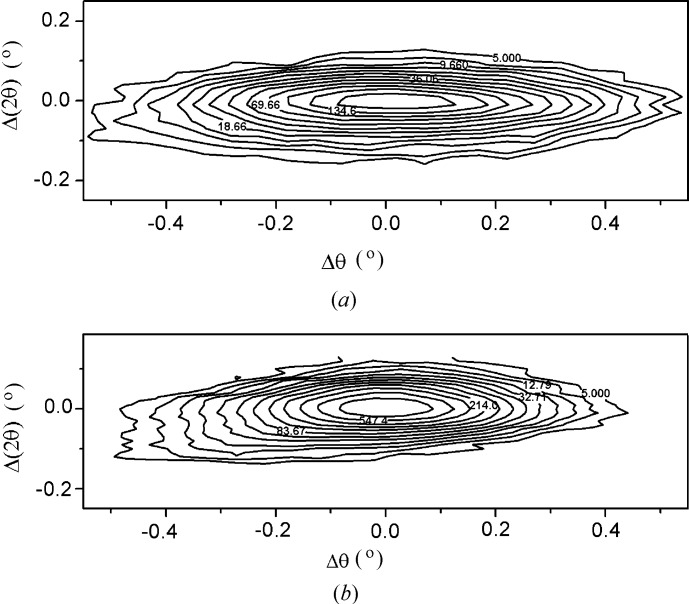
Reciprocal-space maps for (*a*) three-wave diffraction 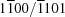 around reciprocal-lattice point 0001 and (*b*) two-wave Bragg reflection 0002.

The intensity distribution around the reciprocal-lattice point of the primary reflection, constructed for the highest intensity three-wave combination 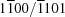, is presented in Fig. 2 ▶(*a*). For comparison, the map for the two-wave 0002 symmetrical reflection is shown in Fig. 2 ▶(*b*). The maps have an ellipsoid shape extended in the direction normal to the diffraction vector. However, for most of the structures, the corresponding θ peaks in the three-wave diffraction are noticeably more broadened than those in the two-wave case (with FWHMs of 1300 and 710′′, respectively). At the same time, the FWHM values of the θ–2θ peaks are approximately the same (230 and 180′′).

Unlike the ϕ-scan peaks, the diffraction peaks of the θ scan have a symmetrical form. For highest perfection layers, the peaks of all three-wave combinations can be approximated with a Gaussian. With increasing dislocation density, the Lorentzian contribution to the peak broadening grows.

The FWHM of the θ-scan peaks strongly depends on the type of the three-wave combination and may vary several-fold between reflections. The peaks corresponding to the three-wave combinations for which the reciprocal-lattice vector **OH**2 is parallel to the surface (Laue reflection) have the largest FWHM (in our notation, these are MP-2, MP-5 and MP-10 three-wave reflections). The narrowest θ peak is observed for the 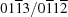 combination, in which the **OH**2 vector has a larger component normal to the surface and a smaller one parallel to it. This behavior is observed irrespective of the absolute values of FWHM.

**Figure 3:**
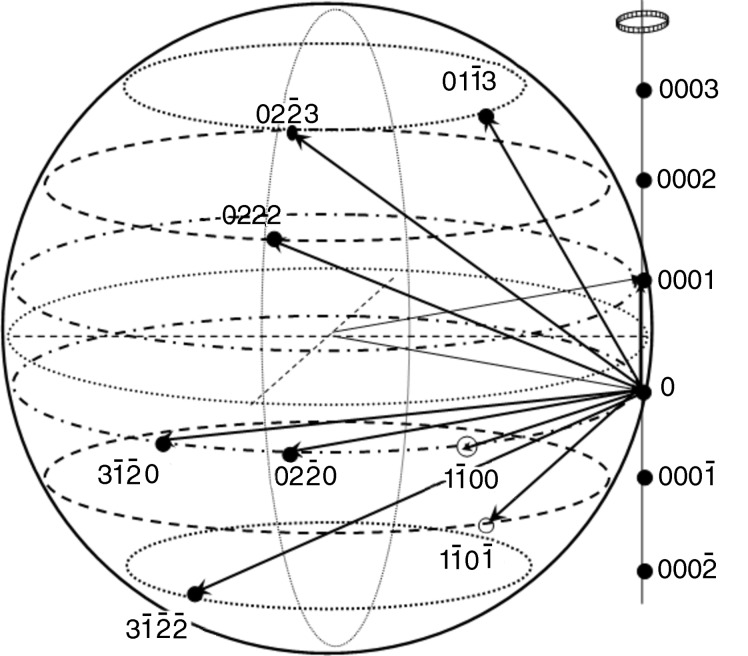
Ewald sphere with the crossing positions of the RLPs of the secondary reflection during the ϕ scan.

Generally speaking, the difference in the peak broadening for different three-wave combinations is determined by the latitude and longitude of the position in which RLP *H*2 of the secondary reflection crosses the Ewald sphere. These positions are shown in Fig. 3 ▶. For all the reflection pairs, we have only three latitude levels corresponding to Miller indices *hk.l* of *H*2 with *l* = 0, 1 (level 1); −1, 2 (level 2); −2, 3 (level 3).

As an example, Table 2 ▶ lists the FWHM values for three GaN films with thicknesses of about 10 µm and different structural perfections. The density of the threading screw and edge dislocations varies within the range 1 × 10^8^–5 × 10^9^ cm^−2^. The data are sorted by the latitude level. It is seen that the narrowest peaks are observed for level 3 and the broadest peaks for level 1.

The θ-scan peaks demonstrate a strong dependence on the structural perfection. Their FWHM values vary within a very large angular range (500–7000′′ for different layers). For the most perfect layer with a dislocation density of ∼10^7^ cm^−2^ (according to transmission electron microscopy data), the FWHM of the θ-scan peaks is nearly the same for all the three-wave combinations (530–560′′). Certainly, the reflections belonging to latitude level 1 are the most sensitive to the dislocation density (see Table 2 ▶). Unfortunately, the available data give no way of establishing any direct correlation between the θ broadening and the density of individual dislocation ensembles.

**Figure 4:**
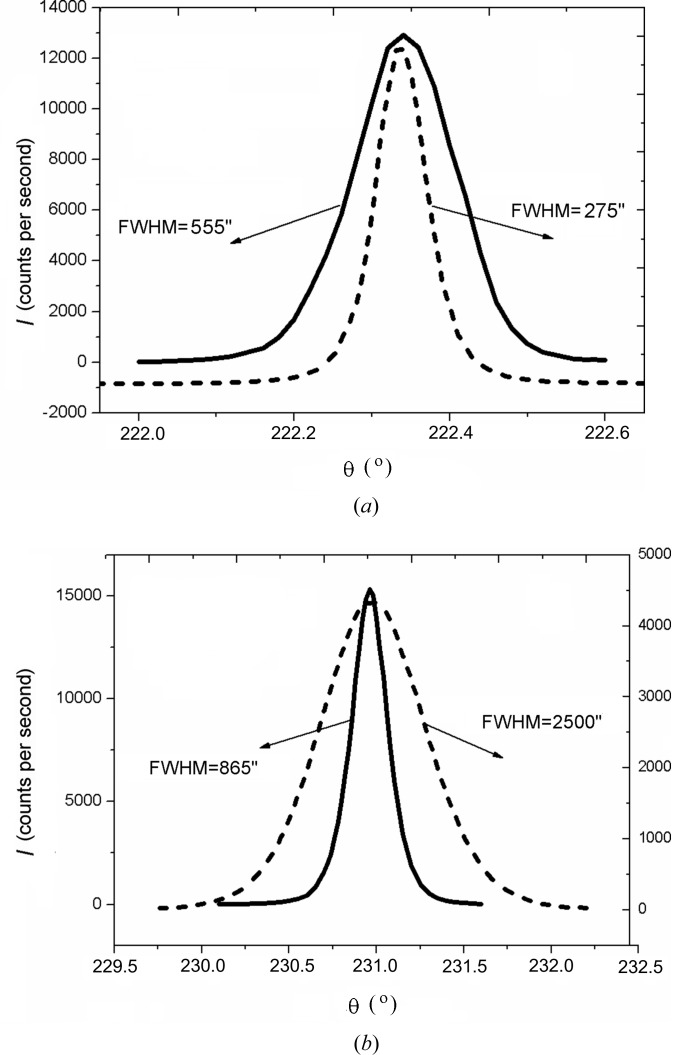
Diffraction peaks of the θ scan in (*a*) the two-wave case (Bragg reflection 0002) and (*b*) the three-wave case (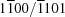 combination) for GaN layers with randomly distributed dislocations (solid lines) and rectilinear threading dislocations (dashed lines).

Experimental data also demonstrate a dependence of the broadening of θ-scan peaks on the type of the dislocation structure. For films with a random dislocation distribution, the FWHM values of θ scans of all the multiple peaks are commonly smaller than those for films with rectilinear threading dislocation ensembles. However, the θ peaks of the two-wave diffraction (in both the Bragg and the Laue geometries) may be considerably broader in the first case and, hence, the density of dislocations may be higher than that in the second case. An example is shown in Fig. 4 ▶.

Some specific features of the three-wave diffraction pattern can be explained from the geometric standpoint. The broadening of the peaks may be determined by two factors: the shape of the RLP of the secondary reflection and the path along which this RLP crosses the Ewald sphere. Rossmanith (2000 ▶, 2007 ▶) considered the ‘reciprocal-lattice spheres’ (for the spherical crystallites) on the Ewald sphere. By contrast, the RLPs due to dislocations are mainly disc shaped for the diffraction pattern of epitaxial layers. The RLP shape depends on the anisotropy of displacement fields around defects and on the mutual orientation of the displacement and diffraction vectors. For randomly distributed dislocations, the discs are normal to the diffraction vector. For rectilinear dislocations, the discs are normal to the direction of the dislocation lines (parallel to the surface in the case of a prevalent density of threading dislocations). The second factor is described above.

**Figure 5:**
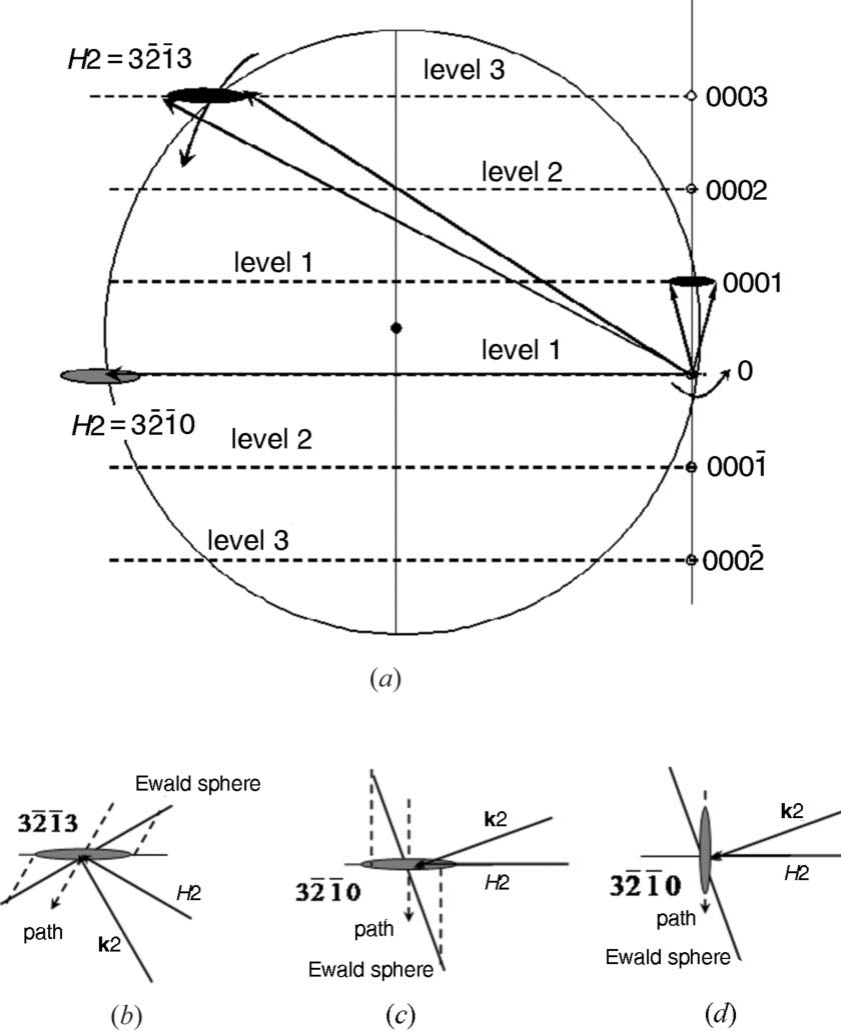
Geometry of the θ scan in the reciprocal space. (*a*) Section of the Ewald sphere with reciprocal-lattice point *H*2 of the secondary reflection lying at the latitude level 3 and at level 1. (*b*), (*c*) and (*d*) Enlarged parts of the Ewald sphere showing the path of the RLP *H*2 during the θ scan. The circles in (*b*), (*c*) and (*d*) are replaced with tangents.

During the θ scan, the reciprocal-lattice vectors rotate about the axis normal to the scattering plane of the primary reflection. This situation is illustrated by Fig. 5 ▶. When we have the predominant density of rectilinear dislocations threading normal to the surface (the intensity distribution is extended in a direction parallel to the surface), the path length of the RLP disc crossing the Ewald sphere strongly depends on the angles between the following three directions: wavevector **k**_2_, reciprocal-lattice vector **H**2 and the normal to the surface (*n*). This length is the larger, the lower the latitude of the RLP *H*2 on the Ewald sphere [see Figs. 5 ▶(*b*) and 5 ▶(*c*) for levels 3 and 1, respectively].

At the same time, comparison of Figs. 5 ▶(*c*) and 5 ▶(*d*) shows that the path length for the RLP disc normal to the diffraction vector (randomly distributed dislocations) may be shorter than that for the disc parallel to the surface. This circumstance partly accounts for the difference in broadening between the θ peaks in Fig. 4 ▶.

However, we cannot explain in this way the fact that the FWHM values of the θ peaks in the three-beam case are larger than those for the symmetrical two-beam diffraction. As follows from Fig. 5 ▶, this means that, during the θ scan, we detect the intensity when the RLP *H*1 leaves the Ewald sphere.

### 4.3. Integrated intensity

The integrated intensities of the three-beam reflections, measured as described above, are listed in Table 3 ▶ for several samples under study. The peak belonging to the 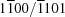 (MP-5 in our notation) combination on the Renninger diagrams has the highest intensity for all the layers, irrespective of their thickness. Therefore, the integrated intensities are given in Table 3 ▶ relative to the intensity of the strongest reflection. According to theoretical calculations based on the kinematic approach (Bläsing & Krost, 2004 ▶), the rest of the three-wave combinations can be divided into three groups: (i) MP-3 and MP-8 (intensity 0.56), (ii) MP-1 and MP-7 (0.22), and (iii) the remaining combinations with lower intensities (0.11). The experimental data are in qualitative agreement with the results of calculations. However, the experimental relative integrated intensities are commonly lower (and for some layers noticeably lower) than the calculated values. Apparently, different three-wave combinations have different penetration depths and their intensities differently depend on the layer thickness. However, the radiation penetration depth is limited by the photoelectric absorption. This limit can be estimated at 5–6 µm for GaN and ZnO and 13 µm for AlN (tenfold decrease in intensity). Of course Rossmanith’s theory was concerned with totally different objects. The difference is that Rossmanith’s studies considered high-perfection spherical crystallites, whereas our objects of study are epitaxial layers with directed systems of dislocations. However, we only compared the integral intensities, which must be identical in the kinematic approximation, irrespective of a particular structure for sufficiently thick samples. The structures represented in Table 3 ▶ have layers with thickness substantially exceeding the penetration depth (except for the ZnO layer). Therefore, we have to assume that there are some other contributions that differently affect the intensities of different three-wave reflections. A possible contribution of this kind is that from the secondary extinction, which should be taken into account by considering the diffraction in thick distorted layers but is very difficult to estimate.

At the same time, it is worth noting that the absolute values of the integrated intensity for the highest intensity MP-5 reflection are rather close to each other for layers with different degrees of structural perfection. This means that the diffraction in these layers satisfies the kinematic limit (their structural imperfection is such that the diffraction in these layers is of the kinematic type). The only exception is the film separated from the substrate (free-standing layer). In this case, the absolute values of the integrated intensity of the three-wave reflections are lower. This sample has a higher degree of structural perfection and the diffraction in this sample is apparently not purely kinematic any longer, with dynamic effects (primary extinction) coming into play.

### 4.4. Forbidden 0001 reflection

**Figure 6:**
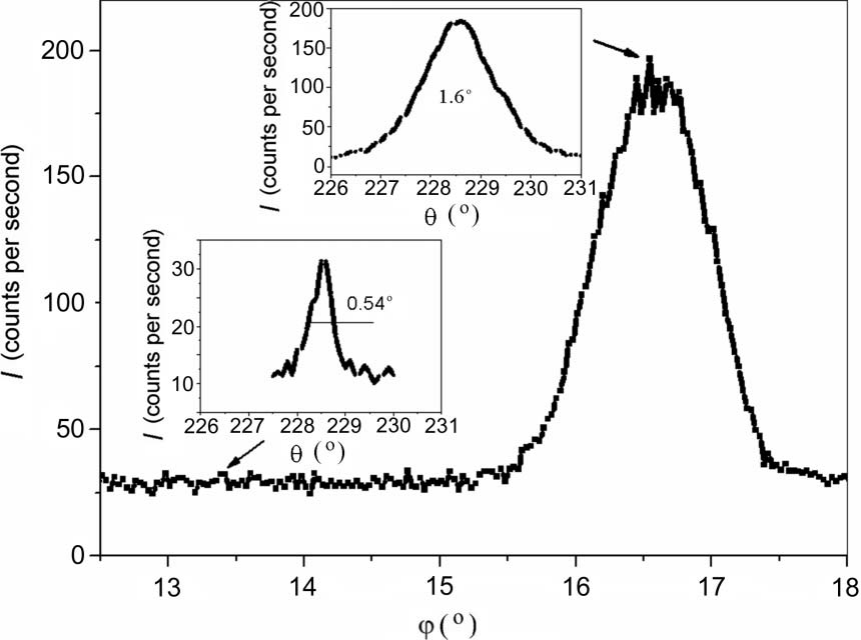
Part of the Renninger scan with 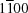/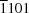 three-wave peaks for the AlGaN layer, and θ peaks measured in the three-wave peak position and at the background level (insets).

If we compare Renninger diffraction patterns measured for the GaN, AlN and AlGaN layers, we see that the background intensity between the three-wave peaks for AlGaN is twice that for GaN and AlN. This additional intensity is the nonzero pure reflection 0001 forbidden for wurtzite structures (structural factor is 0). Its appearance indicates a partial ordering of the AlGaN solid solution. The θ-scan peaks measured at the azimuthal positions corresponding to the three-wave peak and to the 0001 reflection confirm this conclusion (Fig. 6 ▶). It is noteworthy that the 0001 peak is remarkably narrower than the three-wave θ peak.

## 5. Conclusion

Three-wave diffraction was studied in detail in III–nitride and ZnO epitaxial films with different thicknesses and structural perfections. These samples were preliminarily analyzed by two-wave diffraction and parameters of their defect structure were determined. In the three-wave version, the Renninger diagrams were measured for the 0001 primary reflection forbidden for the wurtzite structure, and the intensities and the angular widths of both ϕ- and θ-scan three-wave peaks were analyzed. These experimental data enabled us to reach a conclusion about specific features of the three-wave diffraction pattern in distorted epitaxial layers. Some of these features were accounted for in terms of the reciprocal space. With the disc-shaped intensity distribution and the position of RLPs on the Ewald sphere taken into account, it was shown that the broadening of the θ-scan peaks varies between different three-wave combinations and depends on the type of the dislocation distribution. However, some facts observed in the experiment cannot be described from this standpoint, especially the larger FWHM of θ scans in three-wave diffraction in comparison with those of the two-wave type. The question as to why the relative integrated intensities are noticeably smaller than those predicted by the theory for the kinematic limit also remains unanswered.

The main reason for application of three-wave diffraction to strongly distorted layers was to find a new opportunity for determining the defect structure parameters by using the simplest geometry of the symmetric Bragg reflection. Unfortunately, the available data give no way of coming to an unambiguous conclusion about the dislocation ensembles (vertical edge, screw or horizontal dislocations) making the largest and smallest contribution to the broadening of certain three-wave reflections. Further experimental studies of a wider set of samples with different structural characteristics are required for this purpose.

## Figures and Tables

**Table 1:** Positions of the three-beam peaks on the Renninger diffraction pattern

		ϕ position (°)		
Number	Reflections	GaN	AlN	ZnO
MP-1 (1, 2)†	02 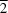 3/0 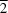 2 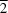	6.48	3.66	7.25
MP-2 (4, 1)	3 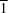 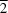 0/ 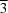 121	7.37	9.01	6.155
MP-3 (3, 3)	01 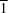 3/0 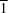 1 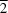	10.35	7.13	10.35
MP-4 (2, 5)	12 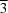 3/ 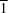 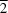 3 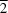	11.04	5.96	12.85
MP-5 (5, 6)	1 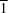 00/ 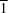 101	16.38	16.81	16.06
MP-6 (6, 8)	02 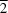 2/0 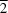 2 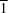	19.91	18.47	20.64
MP-7 (7, 11)	02 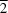 1/0 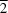 20	25.66	24.67	26.41
MP-8 (8, 10)	1 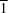 0 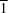 / 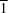 102	26.23	27.29	26.00
MP-9 (9, 9)	3 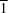 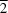 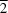 / 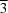 123	27.17	27.75	25.37
MP-10 (10,12)	3 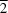 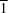 0/ 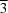 211	29.15	29.20	27.94
MP- - (–, 4)	13 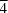 1/ 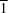 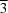 40			11.97
MP- -- (–, 7)	4 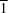 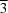 0/ 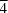 131			20.24

† The numbers are given for GaN and those in parentheses for AlN and ZnO.

**Table 2:** FWHM of the θ-scan peaks (arcseconds), hydride vapor phase epitaxy growth, *t* ≃ 15 µm

Structure	Dislocation density(10^8^ cm^−2^)ρ_vsc_/ρ_ved_/ρ_horiz_ †	Latitude									
		Level 3				Level 2		Level 1			
		01 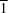 3	02 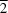 3	3 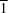 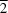 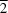	12 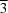 3	1 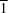 0 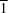	02 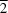 2	1 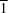 00	02 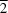 1	3 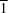 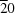	3 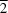 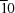
GaN–sapphire	0.34/7.0/0.45	585	890	730	960	975	1080	1060	945	1480	1160
GaN–sapphire	3.0/4.5/1.2	730	1025	995	1100	1215	1110	1190	1080	2060	1710
GaN–sapphire	–/12/45	1280	1490			1940		3160		4670	2960

† ρ_vsc_, ρ_ved_ and ρ_horiz_ are the dislocation densities for vertical screw, vertical edge and horizontal fragments of dislocations correspondingly estimated from analysis of FWHMs of diffraction peaks of two-wave diffraction.

**Table 3:** Integrated intensity of the three-wave reflections

	*t*(µm)	Dislocation density(10^8^ cm^−2^) ρ_vsc_/ρ_ved_/ρ_horiz_)	MP-1	MP-2	MP-3	MP-4	MP-5	MP-6	MP-7	MP-8	MP-9	MP-10
GaN–SiC	8	1.2/13/0.9	0.013	0.04	0.075		1		0.072	0.14	0.006	0.04
GaN–SiC–Si	10	–/6.5/7.1	0.12	0.06	0.46	0.04	1	0.09	–	0.48	0.07	0.08
GaN–sapphire	30	–/90/25	0.045	0.056	0.31	0.011	1	0.041		0.31		0.034
GaN free state	300	0.3/–/–	0.035	0.049	0.21	–	1	–	–	0.29	–	0.015
ZnO	4	<0.01/8/0.1	0.013	0.04	0.08	0.033	1	0.045		0.16		0.024
Theory			0.22	0.11	0.56	0.11	1	0.11	0.11	0.56	0.11	0.11
